# The 5th Canadian Symposium on Hepatitis C Virus: We Are Not Done Yet—Remaining Challenges in Hepatitis C

**DOI:** 10.1155/2016/7603526

**Published:** 2016-10-24

**Authors:** Nicholas van Buuren, Lorraine Fradette, Jason Grebely, Alexandra King, Mel Krajden, Sonya A. MacParland, Alison Marshall, Sahar Saeed, Joyce Wilson, Marina B. Klein, Selena M. Sagan

**Affiliations:** ^1^Department of Genetics, Stanford University School of Medicine, Stanford, CA, USA; ^2^Centre de Recherche du Centre Hospitalier Universitaire de Montréal (CHUM), Montreal, QC, Canada; ^3^The Kirby Institute, UNSW, Sydney, NSW 2052, Australia; ^4^Simon Fraser University, Burnaby, BC, Canada; ^5^British Columbia Centre for Disease Control, Vancouver, BC, Canada V5Z 4R4; ^6^University of British Columbia, Vancouver, BC, Canada V6T 1Z4; ^7^Multi-Organ Transplant Program, University Health Network, University of Toronto, Toronto, ON, Canada; ^8^Chronic Viral Illnesses Service, Division of Infectious Diseases and Research Institute of the McGill University Health Centre, Montreal, QC, Canada; ^9^Department of Microbiology and Immunology, University of Saskatchewan, Saskatoon, SK, Canada; ^10^Department of Microbiology and Immunology, McGill University, Montreal, QC, Canada H3A 2B4

## Abstract

Hepatitis C virus (HCV) affects approximately 268,000 Canadians and results in more years of life lost than any other infectious disease in the country. Both the Canadian Institutes of Health Research (CIHR) and the Public Health Agency of Canada (PHAC) have identified HCV-related liver disease as a priority and supported the establishment of a National Hepatitis C Research Network. In 2015, the introduction of new interferon- (IFN-) free therapies with high cure rates (>90%) and few side effects revolutionized HCV therapy. However, a considerable proportion of the population remains undiagnosed and treatment uptake remains low in Canada due to financial, geographical, cultural, and social barriers. Comprehensive prevention strategies, including enhanced harm reduction, broader screening, widespread treatment, and vaccine development, are far from being realized. The theme of the 2016 symposium, “We're not done yet: remaining challenges in Hepatitis C,” was focused on identifying strategies to enhance prevention, diagnosis, and treatment of HCV to reduce disease burden and ultimately eliminate HCV in Canada.

## 1. Introduction

Hepatitis C virus (HCV) affects approximately 268,000 Canadians and causes more years of life lost than any other infectious disease in the country [1]. The Canadian Institutes of Health Research (CIHR) and the Public Health Agency of Canada (PHAC) have identified liver disease due to HCV as a major public health problem. In 2015, new interferon- (IFN-) free therapies with high cure rates (>90%) and fewer side effects than previous treatments were approved by Health Canada. Currently, government funding supports these therapies for HCV-infected individuals who have moderate to severe fibrosis (F2 and above); but it is anticipated that, in the future, all HCV patients will need to be engaged in care to assess their treatment needs. However, a considerable proportion of the population remains undiagnosed and treatment uptake remains poor due to financial, geographical, cultural, and social barriers [2]. As a result, comprehensive prevention strategies including enhanced harm reduction, broader screening, widespread treatment, and vaccine development are far from being realized. This commentary reviews the 2016 Canadian Symposium on Hepatitis C virus entitled “We're not done yet: remaining challenges in Hepatitis C” that focused on identifying strategies to enhance prevention, diagnosis, and treatment of HCV using the newly approved IFN-free therapies. The conference included discussions on the need for and current state of development of an HCV vaccine, which could help realize the elimination of HCV in Canada.

## 2. The Canadian Network on Hepatitis C (CanHepC)

In 2014, CIHR and the PHAC with the help of nongovernmental (e.g., the Canadian Liver Foundation), industry, and private organizations announced their intention to support a new National Hepatitis C Research Network with a broadened mandate. The intent was to build on the success of their prior investment in the National CIHR Research Training Program in Hepatitis C (NCRTP-HepC). CanHepC was created in July 2015, to expand the NCRTP-HepC training mandate into a National Collaborative HCV Research Network dedicated to translational research. CanHepC links over 60 researchers and knowledge users (community members, community-based organizations, and policy and decision makers) from across Canada as well as international partners. The overarching goal of this network is to improve HCV prevention and health outcomes of Canadians and have a global impact by building innovative research capacity that translates evidence into policy and policy into practice, by focusing on the themes of* prevention*,* treatment*, and* outcomes*.

CanHepC is built around research cores spanning the four CIHR health research pillars: Biomedical Research, Clinical Research, Health Services Research, and Social, Cultural, Environmental, and Population Health Research. Identified priorities include the development and testing of HCV vaccines, increased testing and identification of infected individuals among vulnerable populations, development of strategies to increase access and adherence to treatment, and generation of evidence-based models of care as well as assessment of HCV treatment cost-effectiveness. The four research cores are tightly integrated through shared resources and infrastructure, which is overseen by the CanHepC Steering and Scientific Committees. In addition, Knowledge Translation and Implementation core teams bring representatives from affected communities together with policy makers to help inform how research can address community needs so that the health care system can improve its access, reach, and effectiveness.

CanHepC continues to run the NCRTP-HepC training program and will continue to organize the annual Canadian Symposium on Hepatitis C Virus. Graduate students and postdoctoral fellows are mentored by one of the many CanHepC mentors from each of the four research cores. In addition to their own HCV-related research projects, trainees participate in weekly national seminars and journal clubs, participate in knowledge translation with the community, attend and participate in National and International HCV conferences, and participate in grant writing exercises. Several past trainees are now young investigators and mentors within the CanHepC program highlighting how successful the research training program has been.

In addition to training the next generation of researchers, CanHepC is uniquely positioned to support the transdisciplinary research and collaboration that will be needed to eliminate HCV in Canada. It was with this new mandate that the CanHepC network hosted the 5th Canadian Symposium on Hepatitis C Virus on February 26, 2016.

## 3. The 5th Canadian Symposium on HCV (CSHCV)

Over the past 5 years, the Canadian HCV research community has facilitated HCV research translation in Canada by organizing the annual CSHCV [1, 4, 5]. In response to feedback from community groups and the first four symposia, the specific aims of the 5th CSHCV were

1. to discuss approaches to increase prevention, diagnosis, treatment uptake, and expansion of care to difficult to reach populations with the goal of eliminating HCV in Canada,
2. to facilitate transdisciplinary knowledge exchange and collaborations between Canadian trainees, established researchers, healthcare practitioners, health policy makers, and community-based groups working in the field of HCV,
3. to disseminate symposium findings to support practice change, community awareness, harm reduction, and treatment policy development.

A one-day symposium was held on Feb 26th, 2016, in Montreal, Quebec, Canada. The theme of the meeting, “We're not done yet: remaining challenges in Hepatitis C,” reflected the widespread perception that we have tackled the HCV epidemic with recent advances in drug development and that further research/training in this field may no longer be required. The meeting challenged this perception by asking the following questions of our invited speakers:* (1) What have we learned so far? (2) What remains to be done? (3) What is the best path forward to reach the ultimate goal of eliminating HCV as a major public health issue in Canada?* The meeting focused on identifying strategies to enhance prevention, diagnosis, and treatment of HCV using the new highly effective therapies and included discussions on the need for and current state of development of an HCV vaccine, which in conjunction with new therapies could lead to HCV elimination. The symposium brought together transdisciplinary research scientists, clinicians, nurses, community health workers, patient advocates, and public health officials to discuss future HCV research and care priorities in Canada. All presentations from this and prior symposia are available on the CanHepC YouTube channel (https://www.youtube.com/channel/UCUgCySYhpXIUuquiaQS_rGJw).

### 3.1. Clinical Sciences: Real World Experiences with DAAs

As a result of tremendous efforts in drug discovery and development, there are currently seven approved all-oral HCV direct-acting antiviral (DAA) regimens and an eighth combination pending approval [6, 7]. Although these DAAs were highly successful in clinical trials, the prescribers and patients that participate in these trails are a very narrowly selected population. This raises the question of the efficacy of DAAs administered in real world situations. This question was highlighted by Dr. Nelson (University of Florida, Gainesville, USA) in his talk entitled “Targeting HCV: What Have we Learned from Real World Roll Out of DAAs?” [6]. He described data from the HCV-target multicenter, a prospective, observational cohort of 3000 patients in the United States that suggests that real world DAA efficacy is generally consistent with phase II/III clinical trial data [8]. The data suggests that the unmet need at this point is in treating genotype 3, as the response is suboptimal for these patients [7]. In addition, the data suggests that 8-week regimens for ledipasvir/sofosbuvir have been underutilized and that HCV RNA detection at week 4 of treatment is not indicative of treatment failure and should not be used to guide the duration of treatment [8]. In terms of safety profiles, DAA regimens have very low discontinuation rates (less than 2%) and reports of adverse events during all-oral regimens are much lower than those observed with pegylated-interferon (PEG-IFN) and ribavirin (RBV) [8]. He concluded by stating that the main limitation of real world studies is the inability to compare between the various treatment regimens, although the Patient-Centered Outcomes Research Institute (PCORI) will soon start a head-to-head comparison of several treatment regimens in the United States [9–11].

For the vast majority of patients, the development of resistance to DAA therapy is not a major concern. However, despite the great success of DAAs, when individuals fail, they may develop cross-resistance to multiple drugs [12]. This raises a public health concern because these individuals may then transmit this resistance. This was addressed by Dr. Harrigan (BC Centre for Excellence in HCV/AIDS, University of British Columbia, Vancouver, Canada) in his talk entitled “Will HCV Antiviral Resistance Matter?” [13]. He discussed several studies that examined treatment failure in individuals with baseline resistance associated variants (RAVs). For example, phase II studies of ombitasvir (OMV)/paritaprevir (PTV)/ritonavir (RTV) + dasabuvir (DSV) showed that there is a 2-fold higher failure rate in patients with a baseline HCV NS3 Q80K mutation (88% versus 94%) although these patients still had a high likelihood of achieving a sustained virologic response (SVR) [14]. Dr. Harrigan raised the point that screening individuals for baseline NS3 RAVs prior to therapy initiation could prevent treatment failure and the transmission of resistance.

The theme of real world clinical outcomes was continued by Dr. Samadi-Kochaksaraei (University of Calgary, Calgary, Canada) [15] and Dr. Huchet (Clinique Médicale l'Actuel, Montreal, Canada) [16]. Dr. Samadi-Kochaksaraei described a Canadian cohort study carried out in Calgary, where 362 HCV positive patients were treated with second generation DAAs (349 HCV-monoinfected, 12 HIV/HCV-coinfected, and 1 HCV/HBV-coinfected). Similar to the clinical trial reports, therapy was well tolerated and highly effective, and over 92% of patients treated achieved an SVR [15]. Dr. Huchet described a similar study carried out in Montreal [16]. Two-hundred and forty-nine patients were included in this study. The majority of patients were treatment naive and infected by HCV genotype 1. Forty percent of patients were cirrhotic and 25% were HIV coinfected. In this cohort, 85% of patients achieved SVR. Cirrhotic patients were less likely to achieve SVR (78% for cirrhotic versus 91% for noncirrhotic patients, *p* = 0.02). As such, in this cohort, SVR rates were reduced by 15–20% when compared to clinical trial data. This was attributed to the high proportion of late stage liver disease in this cohort which likely requires more prolonged treatment regimens that are tailored to baseline resistance profiles [16]. While these findings confirm that achieving a SVR can be more difficult in cirrhotic patients, the overall rates of SVR in patients, even those with late stage disease, provide renewed hope for those affected.

### 3.2. HCV Care and Treatment: The Evolving Role of the Hepatology Nurse

Continuing the theme of real world experiences with DAAs, Hirsch (Registered Nurse-Nurse Practitioner, Nova Scotia Health Authority, Halifax, Canada; Member of Canadian Association of Hepatology Nurses) presented the hepatology nurse viewpoint [17]. Hirsch opened by summarizing results from a 2015 National survey conducted by the Canadian Association of Hepatology Nurses (CAHN). Among 94 CAHN members, 78% were >40 years, 35% received salary support from industry, and more than half (60%) expressed concern about job security and position sustainability [18]. Referring to the HCV treatment cascade [19], Hirsch highlighted that during the era of IFN-based treatment, hepatology nurses were primarily involved in the HCV treatment stage. This led to the development of hepatitis nurse specialists who were responsible for providing patient education and support and monitoring treatment efficacy and side effects. Some hepatology nurses branched into areas of primary care and addictions while others with specializations in liver disease prescribed treatment. Today, nurses play an increasing role in working with marginalized communities to facilitate HCV diagnoses and access to care. The development of IFN-free regimens eliminates much of the pretreatment work-up and past treatment contraindications, providing the opportunity to decentralize care to nonspecialized physicians and nurses, particularly in rural Canada. Further, Hirsch underscored that the current HCV model of care is too siloed. An alternative care paradigm places nurses at various access points including an increased role among high-risk population groups to better facilitate HCV identification and diagnosis (e.g., onsite point-of-care testing led by nurses). Hirsch added that standard testing algorithms for people with addictions, sex-trade workers, and prisons are needed, as well as testing guidelines for the baby-boomer cohort. Lastly, Hirsch emphasized that, in addition to treatment initiation, nurses will become increasingly more involved with providing hepatology care to individuals with advanced liver disease.

### 3.3. Biomedical Sciences: The Current State of HCV Vaccine Development

Although we have access to highly effective DAAs for HCV treatment, Dr. Cox (Johns Hopkins University, Baltimore, USA) highlighted that no infectious disease has ever been eradicated without effective prevention measures [20]. Additionally, eradication of HCV with DAAs alone is subject to complications such as drug resistance, poor identification of infected persons, susceptibility to reinfection, and patients often not seeking health care until the presentation of end stage liver disease (ESLD). For these reasons, the development of an HCV vaccine is of the utmost importance. But, is a vaccine even possible? Several patients that have cleared their primary infection with HCV have gone on to become chronic carriers upon reinfection. Additionally, patients cured using DAAs can still become reinfected, suggesting a lack of protective immunity. However, Dr. Cox presented data that suggests that most patients that clear an acute HCV infection have a much higher clearance rate upon reinfection [21]. Patients that have cleared a primary HCV infection demonstrated lower levels of viremia and shorter viremic periods upon secondary exposure [21]. These data suggest that protective immunity does exist, at least in some individuals. Therefore, a potential goal of a vaccine should be to mimic protective immunity.

Dr. Cox is currently running a phase II clinical vaccine trial entitled “Vaccine Is Prevention (VIP) in the Baltimore Before and After Acute Study of Hepatitis (BBAASH),” cohort of intravenous drug users. Cohorts such as this are good avenues for testing vaccines as the HCV infection incidence is very high (~30%) and many in the cohort enter negative for HCV antibodies and viral RNA. Clinical trials conducted within these marginalized populations are extremely challenging, but compliance can be maintained by incentives including drug counseling, clean injection materials, access to opioid substitution therapies, and financial gain. This phase II clinical trial investigates a T-cell vaccine developed by Swadling et al. that makes use of the HCV nonstructural proteins and is composed of a prime and boost component [22]. Although data on efficacy are not yet available, Dr. Cox demonstrated phase I results that indicated that the vaccine was safe and elicited a robust T-cell response in all participants (efficacy data will be available later this year) [22]. It will be very exciting to learn whether this vaccine induces long term T-cell memory that protects against chronic HCV infection.

As highlighted above, the successful HCV vaccine will likely mimic the immune response generated in patients that resolve HCV following acute infection. The identification of immune signatures that correlate with clearance is a major focus of Dr. Shoukry's (Université de Montréal, Montreal, Canada) research [23]. Identification of patients with acute HCV infection is a challenge as symptoms are typically mild and therefore most people do not seek health care during this time point. The Montreal Hepatitis C Cohort (HepCo) overseen by Dr. Julie Bruneau has enrolled nearly 140 people who inject drugs (PWID) that are HCV negative but are at risk for contracting the virus. To date, this cohort has identified eleven previously HCV negative patients that acquired HCV while being monitored. Of these eleven patients, six spontaneously cleared their infections, while five became chronically infected. Blood samples from patients within the HepCo were profiled by deep sequencing to learn which immune response genes are turned on or off in patients that cleared HCV. Notably, innate immune signatures were slightly higher, and B-cell signatures were slightly lower, in the population of resolvers during the early acute phase of infection [23]. As more patients are identified with acute HCV, this research will continue to provide clues as to the molecular signature that underlies HCV protective immunity.

The development of highly effective HCV DAAs over the past 25 years was and continues to be the collaborative achievement of hundreds of scientists spread across academia and industry. With so many DAAs already in the pipeline, many basic researchers are trying to establish new research avenues. The research in Dr. Lamarre's (Université de Montréal, Montreal, Canada) laboratory aims to develop broad-spectrum antivirals that could target an array of positive-strand RNA viruses. Dr. Lamarre's laboratory has performed proteomics studies on the HCV nonstructural proteins and identified a number of fascinating cellular proteins that not only are required for HCV infection but also mediate infectivity of other positive-strand RNA viruses [24]. In his presentation, Dr. Lamarre highlighted one such protein, called HSD17B12, which is essential for HCV assembly and egress. This protein appears to decrease the number of lipid droplets in HCV-infected cells and is essential for proper assembly of the membranous web (sites of HCV replication within infected cells) [25]. Many positive-strand RNA viruses replicate their genomes within a membranous web structure that is formed during infection, making it an excellent target for antiviral intervention. It is important to note that knockdown of the host protein HSD17B12 had no impact on cell viability, indicating that targeting this protein* in vivo* may be safe. This protein is therefore one potential host target that could be developed as a broad-spectrum antiviral. In a time where many researchers are looking to move on from HCV, Dr. Lamarre has found an exciting way to capitalize on our extensive knowledge of HCV in order to have impact on a larger number of pathogens.

### 3.4. Debate: Is an HCV Vaccine Really Needed?

Drs. Wong and Cox had a lively debate centered on the question “Do we need an HCV vaccine?” (in spite of the fact that Dr. Wong, who was asked to argue the “con” side of the debate, conceded to Dr. Cox immediately and confirmed that his actual opinion is that we do need a vaccine) [26]. The debate focused on vaccine versus treatment priority setting.

#### 3.4.1. The Priority Should Be on the Use of the Drugs to Treat HCV-Infected People

Dr. Wong argued that the current priority in the HCV field should be to treat all HCV-infected people and expressed his outrage that not all cirrhotic patients in Canada have been treated at present. He suggested that our efforts should focus on increasing treatment capacity by finding ways to decrease the cost of the drugs and by engaging primary care physicians in treatment delivery. He also stated that we should avoid distractions such as treating people without fibrosis and testing for drug resistance since it is very rare. Dr. Cox countered that we cannot ignore resistance since we should expect to see it in the future and that resistance will become a much larger issue if resistant viruses are transmitted.

#### 3.4.2. HCV Elimination: Do We Need Drugs, Vaccines, or Other Treatments?

Dr. Wong argued that the probability of developing an effective HCV vaccine is low since natural infections by HCV do not induce protective immunity. Dr. Cox countered by highlighting that people who spontaneously clear HCV infections are less likely to be infected again, suggesting that protective immunity is possible. Dr. Cox stated that poor funding of vaccine research in the past has slowed progress but that does not mean that a vaccine is not possible.

The discussion also focused on methods of vaccine testing, which is currently limited to high-risk populations including people who inject drugs (PWID) and a population that is difficult and costly to manage. It was also suggested that because we have such effective HCV treatments we could now consider testing vaccines by infecting vaccinated healthy individuals. Any subjects that fail to be protected could then be cured using DAAs, a strategy that is currently in use for Malaria vaccine testing [27, 28]. However, Dr. Cox pointed out that untreatable HCV is possible even with the excellent therapies we have at hand; thus, we would have to carefully assess the risk that we might infect someone with chronic HCV that we are unable to cure.

Dr. Cox further argued that a vaccine will be required to eliminate HCV. Other diseases, like chlamydia and syphilis, are treatable using inexpensive and highly accessible drugs but have yet to be eliminated. Dr. Wong countered that hepatitis B virus (HBV) infection, a disease for which we have an excellent vaccine, is still a problem. In conclusion both Drs. Wong and Cox agreed that HCV elimination is possible if we can combine a vaccine with the currently available DAAs; however, even with an effective vaccine, HCV elimination will require a global effort and support from organizations such as the Bill and Melinda Gates Foundation.

### 3.5. Behavioural and Social Sciences: Improving Access to Care in Marginalized Populations

**Figure 1:**
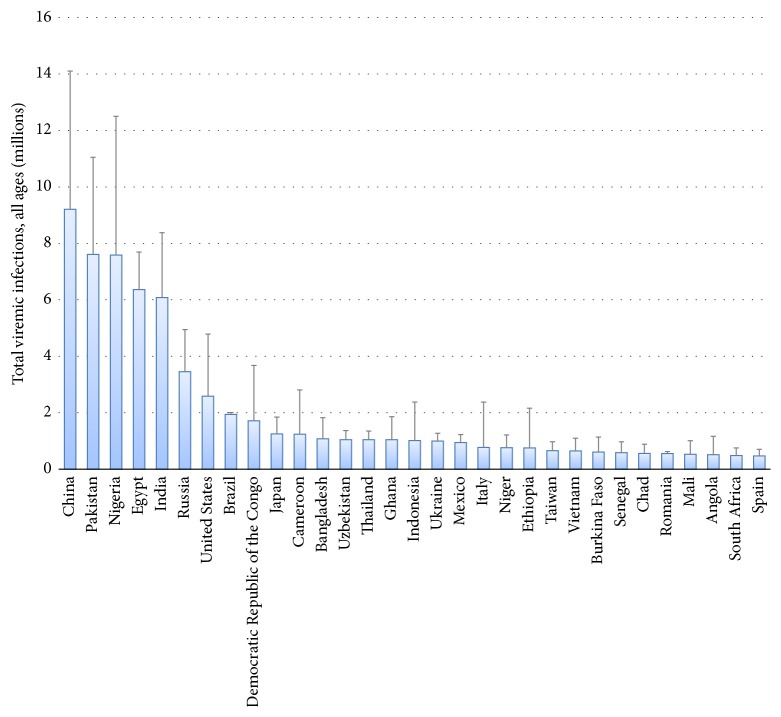
Countries accounting for 80% of the total viraemic HCV infections globally (Gower et al. [3] reproduced with permission from Journal of Hepatology).

In the opening presentation of the Behavioural and Social Sciences session, Dr. El-Sayed (Ain Shams University, Cairo, Egypt) highlighted challenges in the widespread implementation of HCV therapy in low- and middle-income countries [29]. It is estimated that between 80 and 150 million people have chronic HCV infections [3, 30]. However, the majority of HCV-infected individuals reside in low- and middle-income countries [3, 30]. In a systematic review of the global epidemiology of HCV infection, it was demonstrated that 31 countries account for 80% of all infections (Figure 1) [3]. China, Pakistan, Nigeria, Egypt, India, and Russia together account for more than half of all HCV infections globally [3].

The burden of HCV infection in Egypt is particularly high [3]. It is estimated that the incidence of HCV viremia in adults is 10%, and there are 3.9 to 6.9 million people living with chronic HCV infection [3]. Egypt established the National Committee for Control of Viral Hepatitis in 2006 with the goal of developing the following: (1) a national survey to characterize the burden of disease; (2) a national viral hepatitis strategy; (3) an HCV treatment program; (4) implementation of prevention strategies (including community awareness, media campaigns, and improved infection control); (5) clinical research capacity; and (6) strategies for management of advanced liver disease. In 2008, these efforts led to the integration of HCV testing into the National Demographic Health Survey and the publishing of the first national viral hepatitis strategy [31]. In addition, Egypt developed a large-scale treatment program, which was able to treat 350,000 patients with peg-IFN and ribavirin in the first seven years of the program. However, as highlighted by Dr. El-Sayed, other prevention activities, such as infection control, remain somewhat fragmented, with 165,000 new HCV infections still occurring per year (mostly attributed to iatrogenic transmission through transfusion and unsafe medical injection practices).

Between 2011 and 2014, in an attempt to enhance prevention efforts, Egypt sought the advice of the World Health Organization (WHO), the Centers for Disease Control (CDC), and the Pasteur Institute to help in the establishment of an action plan including prevention, screening, care, and treatment. In 2014, Egypt initiated clinical trials with IFN-free DAAs for participants infected with HCV genotype 4. Furthermore, although Egypt is considered a middle-income country, it became one of the first countries in the world to negotiate with Gilead to secure a price of $900 per treatment course for sofosbuvir-based treatments in high volume and developed a well-established infrastructure to deliver therapy.

Despite this, many challenges remain if HCV is to be eliminated in Egypt, including integration of new generic therapies (including issues related to quality assurance programs, voluntary licenses, and prequalification); enhancement of screening and diagnostic testing (including low-cost point-of-care tests); improving access (particularly in remote and rural areas); and addressing stigma and discrimination. Notwithstanding, Egypt is clearly a world leader in addressing viral hepatitis and they provide valuable insights into how to create a working model to achieve national HCV elimination.

There are several populations that experience disproportionately high HCV disease burden worldwide [32]. They include people who use drugs, people with criminal justice system involvement, and those who also have HIV/AIDS. In many countries, Indigenous populations represent some of the highest incidence of HCV infection [33]. Indigenous peoples are among those sometimes known as “key,” “target,” or “marginalized,” but these labels fail to recognize the inherent strengths and resilience of Indigenous peoples or the devastating impacts of colonization and health determinants [34]. Dr. King (Simon Fraser University, Burnaby, Canada) provided information on Indigenous peoples in Canada and HCV by presenting on Indigenous research methodologies [35].

Indigenous peoples in Canada are at a critical junction in the transforming HCV landscape, but also more broadly. The federal government's commitment to the full implementation of the Truth and Reconciliation Commission's Calls to Action, which include references to the United Nations Declaration on the Rights of Indigenous peoples, promotes Indigenous self-determination in the progressive realization of health equity [36]. One way this will be achieved is through HCV research done in collaboration with Indigenous communities, embracing strong Indigenous leadership and representation throughout the research team and process and Indigenous research methodologies are based on Indigenous worldviews and Indigenous Ways of Knowing [37]. Concepts such as relationality, reciprocity, respect, and relevance are critical, contrasting with Western Ways of Knowing, which incorporate such ethical constructs as beneficence, nonmaleficence, autonomy, and confidentiality [38]. Indigenous research done “in a good way” is a sacred endeavor, grounded in ceremony, connected to ancestral wisdom, creating community and contributing to healing. Intentions and processes become as important as the knowledge acquired. Both the individual and the collective are critical perspectives. To successfully incorporate Indigenous research philosophies, an ethical framework is required where different cultures and knowledge systems can engage in dialogue as equal partners. Two such frameworks were presented, including Two-eyed Seeing [39], in which synergies result from simultaneously embracing both Western and Indigenous Ways of Knowing and Ethical Space [40], in which there is recognition of the privilege afforded to Western research methodologies and there is a consequent commitment to “level the playing field.”

Recognizing this fortuitous concurrence of transformation in the HCV landscape and in the broader governance structures impacting Indigenous peoples in Canada, a team of Indigenous people assembled to undertake a decolonized approach (i.e., “by us, for us”) in developing a research framework for Indigenous peoples and HCV. This has become known as* Water Journey: Community-Directed Research Priorities for Indigenous Peoples in Canada and Hepatitis C*; the initial research project was presented by Macklin (Simon Fraser University, Burnaby, Canada) [41]. Four sharing circles (one male, two females, and one two-spirit [42]) were conducted in which Indigenous people with diverse lived HCV experience discussed research priorities, guided by Indigenous elders. The sharing circles were audiorecorded and transcribed verbatim, and then the transcripts were analyzed qualitatively using a grounded theory-based approach. Major themes and subthemes were identified. The results were subsequently validated with other Indigenous communities, helping to build a comprehensive understanding of community-driven research and health priorities for HCV.

Several crosscutting themes were identified, with both individual and system-level factors impacting people's life course with HCV. The key themes include strength and resiliency of First Nations, Inuit, and Métis living with HCV, as well as their families and communities; intersections of multiple HCV risk factors; barriers within the continuum of care; competing priorities in one's life; reduced health literacy; and the importance of transformation and finding purpose along one's healing journey with HCV. Ways forward that honor Indigenous Knowledges and Ways of Knowing were identified [43]. The project highlighted the criticality of Indigenous leadership in setting HCV research priorities, programs, and policies pertaining to First Nations, Inuit, and Métis. Indigenous research methodologies and Ways of Knowing privilege community voice and lived experience. There must be a focus on innovation, self-determination, service integration, cultural safety, wholism, Indigenous values, and the application of strengths-based approaches. The themes identified in the Water Journey project must be taken into consideration across the realms of HCV research, policy, and prevention programs and throughout the continuum of care.

### 3.6. Epidemiology: Strategies to Reduce HCV Burden

Public health aims to improve the health outcomes of an entire population and reduce health inequities among specific groups. To achieve this goal the complete spectrum of the care continuum including prevention, linkage to care, diagnosis, and treatment needs to be utilized [44]. The DAA era has brought optimistic visions of HCV elimination. Elimination is only possible by treating prevalent HCV cases and averting incident cases that arise from new infections and reinfections. The epidemiology and public health section of the symposium followed the theme of the conference “We Aren't Done Yet” and focused on a population at high risk of infection, transmission, and reinfection, PWID. Both keynote speakers provided comprehensive examples of how to reduce new HCV infections by integrating multiple interventions in the care continuum.

Dr. Hickman from Bristol University presented evidence from observational and modeling studies on the impact of harm reduction interventions and how this might impact those prioritized for HCV treatment [45]. Access to opioid substituting therapy (OST) and needle/syringe exchange programs (NSPs) has been shown to significantly reduce HCV transmission [46]. Dr. Hickman presented results from a recent Cochrane Review of 15 studies including 1089 HCV cases over 4262 person years of follow-up time. Pooled results demonstrated that exposure to OST resulted in at least a 54% risk reduction in HCV transmission [47]. Although OST and NSP have been shown to prevent HCV infections, the coverage required to achieve substantial HCV prevalence reductions may be unsustainable (80% compared to the current 50%) and therefore unlikely to be achieved [48]. In contrast, modeling studies suggest that, by scaling up HCV treatment in combination with OST and NSP, HCV prevalence can be reduced substantially and rapidly, especially in settings where chronic HCV prevalence is <20% or <40% [49]. A HCV treatment prioritization analysis using the outcome of net monetary benefits and a willingness to pay threshold of £20,000 (~$38,000 CAN) was performed. These results allowed Dr. Hickman's research group to demonstrate a considerable benefit of prioritizing HCV treatment in PWID with moderate or even mild disease status due to the reduction in transmission risk [50]. The prevention benefit (averting an additional 1-2 HCV infections per treatment) made treating PWID highly cost-effective. Therefore, from a public health perspective, this research supports the argument that, in many settings, PWID should be prioritized regardless of disease stages [50]. The challenge remains that current treatment rates are insufficient to lead to observable changes in HCV prevalence over the next 5–10 years. Caution was addressed that these results were based on modeling studies. The next step would be to design and execute impact evaluation studies in well-characterized PWID populations [45].

Dr. Cox from McGill University expanded on Dr. Hickman's presentation on integrating harm reduction and treatment focusing on the HCV care continuum to advance the overall care of PWID. Dr. Cox emphasized how PWID suffer from addiction that was described as a chronic relapsing brain disease, with limited medical therapies available [51]. In addition, this marginalized population bears multiple comorbid conditions including other mental health disorders and poor social determinants of health. A novel treatment as prevention (TasP+) strategy was described as a combination of primary and secondary prevention that could help both in limiting infection rates and perhaps by increasing the cessation of drugs. This approach utilizes interventions along the HCV care continuum working in parallel to ensure treatment uptake and sustainable cures. Recent modeling studies using data in Montreal were presented, suggesting that early linkage to care leading to initiating DAAs and good adherence could create a remarkable reduction in HCV prevalence in less than 10 years [51]. Dr. Cox highlighted the need to focus on the extreme ends of the care continuum, which meant prevention of new infections and reinfection. Initiatives related to and informed by harm reduction could have the potential to decrease HCV transmission and contribute to a sustainable cure among PWID.

### 3.7. Update on Restriction of Reimbursement of HCV Treatments in Canada

Marshall (UNSW Australia, Sydney, Australia) presented a review of restrictions for reimbursement of DAAs for HCV infection in Canada [52]. With the current list price of 2nd generation HCV DAAs highly prohibitive in Canada, universal coverage is a challenge. The aim of the review was to assess reimbursement criteria for simeprevir (w/PEG-IFN/RBV), sofosbuvir (w/PEG-IFN/RBV or RBV), ledipasvir-sofosbuvir, and paritaprevir-ritonavir-ombitasvir plus dasabuvir (w/ or w/o RBV). First Nations people and Inuit (Non-Insured Health Benefits Program, NIHB) criteria as well as federal prisoner (Correctional Service Canada, CSC) criteria were also collected. Primary outcomes were minimum fibrosis stage required, drug and alcohol use restrictions, HIV coinfection restrictions, and prescriber-type restrictions. From April 2015 to January 2016, information was mainly gathered from publicly available special authorization request forms and drug formularies obtained from online provincial/territorial health ministerial websites. Results highlighted that the majority (82–92%) of jurisdictions restricted reimbursement to moderate fibrosis (≥F2, METAVIR or equivalent). No drug and alcohol use restrictions were found and up to half (50%) of jurisdictions limited prescribing to specialists. Simeprevir or sofosbuvir was not reimbursed for HIV coinfected persons in Quebec (although exceptions can be granted). NIHB and CSC had similar criteria as remaining jurisdictions. Marshall summarized that, although Canada has less HCV DAA reimbursement heterogeneity by jurisdiction than the United States [10], most provinces/territories still apply disease stage restrictions that are not cost-effective [16]. A national HCV strategy in Canada would help reduce interjurisdiction heterogeneity and improve information transparency.

### 3.8. From Research to Action: How to Translate Research into Policy Change

Dr. Krajden (University of British Columbia, Vancouver, Canada) moderated the panel discussion entitled: “From Research to Action: How to Translate Research into Policy Change” [53]. He opened by presenting data from the British Columbia Hepatitis Testers Cohort (BC-HTC) highlighting that the overall mortality for anti-HCV positive individuals was about 2.5-fold higher than in those who tested anti-HCV negative [54]. A key element is that both mortality and related health costs were the result of HCV acquisition risks and the sequelae of chronic HCV infection. HCV acquisition is a surrogate for injection drug use, the most common route of transmission in developed countries. HCV-infected individuals are stigmatized, are often socially and materially deprived, and have a limited political voice. As a result, it has been difficult to obtain the political buy-in for a comprehensive and equity-based Canadian Hepatitis C strategy or action plan. As a society, we also need to support the developing world where most infections result from unsafe injection practices. As such, there is a need to design and deploy nonreusable needles and syringes as part of the prevention tool kit.

HCV is clearly a preventable, and now a curable, disease, which merits a public health approach. Unfortunately, the marketing of treatment-based HCV curability makes it difficult to illustrate that by treatment-based HCV cures we do nothing to address acquisition related morbidity and mortality. Furthermore, the high treatment costs beg for an opportunity of cost discussion! In some cases, the money spent on treatment may be better used to implement prevention and harm reduction measures to support people with addiction and mental health issues which is not socialized in mainstream society. Treatment as prevention (TasP) is another treatment-based approach to limit HCV transmission, but TasP needs to be combined with comprehensive harm reduction programming and be evaluated to ensure that PWID do not get reinfected. For at-risk populations, a syndemic focus will be needed given the comorbidities that affect PWID, the population involved with most onward transmission in Canada.

Dr. Werb (University of California, San Diego, USA, and St. Michael's Hospital, Toronto, Canada) agreed that, at present, HCV treatment is overshadowing the important role of prevention. His talk focused on the value and science of prevention [53]. Prevention needs to be addressed in the context of both national and international policy-making. Canada's new federal government is in the process of creating a new drug policy. This creates an opportunity for researchers to use evidence to help craft the policy. But, the timeframe is short, given that the National Anti-Drug Strategy mandate ends in 2017 and needs to be renewed. The strategy's renewal, combined with other drug legislation, creates a formal process to highlight the issues of prevention, particularly in relation to PWID.

Scientists should try to highlight the importance of supervised injection sites in prevention of HCV transmission. Creation of supervised injection environments in prisons is an outstanding gap. While scientific evidence could help repeal ineffective policies, there are no formal mechanisms for scientists to engage federal civil servants to effect policy. Internationally, there is a planned United Nations (UN) special assembly on the global drug problem. The UN meeting will attempt to set a global drug strategy document to support an international response. Thus far, early drafts of this strategy fail to mention “harm reduction” which needs to be a cornerstone of evidence-based public policy. The Canadian government could and should take a role in promoting the use of “harm reduction” as part of the global science-based drug policy.

Betteridge (Canadian Treatment Action Council, Toronto, Canada) spoke about the “A” words: Aims, Activism, Action, and Advocacy [53]. He highlighted the role of the brand-name pharmaceutical industry in the development of the new, well tolerated, and extremely effective curative DAAs for HCV. He also highlighted the challenges in deciding whether, and in what circumstances, to pay for these expensive treatments. Clearly, there is a need for activism to improve timely, affordable, and equitable access to HCV treatment and care. This typically requires patient advocacy. Approaches include building community representation into clinical and public health research, presenting research evidence to people living with HCV, and supporting community-based health and require that support organizations clarify their needs. This needs to be combined with convening multidisciplinary think tanks that include people living with HCV, writing and signing petitions, letters related to HCV care, treatment, and support including issues like harm reduction and drug policy, preparing summaries of research for policy and decision makers. These messages need to be delivered to the civil servants responsible for healthcare.

Dr. Njoo (Public Health Agency of Canada, Ottawa, Canada) provided a Public Health Agency of Canada (PHAC) perspective highlighting that health services are under Provincial and Territorial Government jurisdiction [53]. While PHAC has responsibility for surveillance, laboratory support, a program funding role (related to PHAC and CIHR), jurisdiction over regulatory, and health product approval (Health Canada) and International response, it lacks the mandate to develop a National Strategy or Action Plan unless there is buy-in from the provinces and territories. However, the federal government does have jurisdiction for the health care and public health service delivery to federal populations, for example, First Nations and Inuit, or people in federal correctional facilities.

PHAC uses collaborative networks such as the Pan-Canadian Public Health Network and Partners in Public Health representing senior officials from all jurisdictions to strengthen public health in Canada. Other standing committees include Communicable and Infectious Disease, Healthy People and Communities, and the Public Health Infrastructure. Together these committees oversee the development of coherent, comprehensive, and collaborative public health policy approaches in Canada. Dr. Njoo recognized the need to use science to support policy and highlighted how there is an equal need to use policy to implement science, particularly in the areas of program delivery and evaluation, using integrated approaches to prevention, and learning how to better leverage and integrate with existing programs or initiatives. He suggested that provincial and territorial approaches need to be leveraged to help engage federal support.

In summary, the panel discussion highlighted the need for a broad and multifaceted approach to translate research into effective communication of findings across the prevention, care, and treatment continuum. This evidence needs to be heard by both mainstream society and decision makers in order to support policy change.

### 3.9. Outcomes of the 5th Canadian Symposium on Hepatitis C Virus

With the approval of highly effective HCV DAA treatments, HCV is now a potentially curable disease. This revolution in HCV treatment is a testimony to what can be achieved by the combined efforts of basic scientists, clinicians, and the pharmaceutical industry. However, it is not enough to eliminate HCV in Canada! The 5th Canadian Symposium on HCV highlighted that, for HCV disease elimination to become a reality, transdisciplinary research across biomedical, clinical, health services, and social, cultural, environmental, and population health domains will need to be translated into policy and practice. This is one of roles of Canada's new CanHepC research network! Yes, there is still a need for an HCV vaccine, but vaccine discovery requires an understanding of host and virus pathogenesis, basic research. The effectiveness and tolerability of the new DAAs create an opportunity to rethink how treatment could be delivered by allied health care professionals including nurses and physicians; Egypt's approach to a National HCV Strategy provides a wake-up call to Canadians regarding what can be achieved, even in low- to middle-income countries, when there is a societal will. In Canada, treatment pricing is a challenge, and to improve treatment access and uptake, this needs to be addressed. Most importantly, HCV elimination in Canada will require engagement and involvement of civil society and in particular the populations affected including PWID, immigrants, Indigenous populations, and Canada's baby boomers. The voice of the affected communities needs to help shape comprehensive solutions that include harm reduction and addiction. Their collective voices need to resonate in Canada's provinces and territories to inform a collective federal, provincial, and territorial HCV elimination strategy or action plan.
